# Investigating the effects of stress on achievement: BIOSTRESS dataset

**DOI:** 10.1016/j.dib.2023.109297

**Published:** 2023-06-08

**Authors:** Çağla Çöpürkaya, Elif Meriç, Elif Berra Erik, Büşra Kocaçınar, Fatma Patlar Akbulut, Cagatay Catal

**Affiliations:** a Department of Computer Engineering, Istanbul Kültür University, Istanbul, Turkey; b Department of Software Engineering, Istanbul Kültür University, Istanbul, Turkey; c Department of Computer Science and Engineering, Qatar University, Doha, Qatar

**Keywords:** Psychological experiments, Stress response, Biosignals, Wearable device

## Abstract

The effects of chronic stress on academic and professional achievement can have a substantial impact. This relationship is highlighted through a dataset that includes questionnaires and physiological data from a group of individuals. The questionnaire data of 48 individuals, the physiological data of 20 individuals during sessions with a psychologist, and the exam data of 8 individuals were analyzed. The questionnaire data collected includes demographic information and scores on the TOAD stress scale. Physiological data was captured using the Empatica e4, a wearable device, which measured various signals such as blood volume pulse, electrodermal activity, body temperature, interbeat intervals, heart rate, and 3-axis accelerometer data. These measurements were taken under different stress conditions, both high and low, during therapy sessions and an exam respectively. Overall, this study significantly contributes to our understanding of how stress affects achievement. By providing a large dataset consisting of questionnaires and physiological data, this research helps researchers gain a better understanding of the complex relationship between stress and achievement. It also enables them to develop innovative strategies for managing stress and enhancing academic and professional success.

**Specifications Table**

**Table:** 

Subject	Computer Science, Data Science, Health and medical sciences
Specific subject area	Stress detection, Signal classification, Signal Processing
Type of data	Signals Survey Form
How the data were acquired	In order to select the most suitable individuals, a survey was conducted to gather data on their demographics, anxiety histories, and stress levels. The survey consisted of two parts that participants were asked to complete. The first part included personal questions, while the second part consisted of the TOAD's Stress Scale questions, comprising a total of 34 questions. Following the completion of the survey, participants were given the opportunity to participate in a stress-inducing session lasting 15 minutes, facilitated by an experienced psychologist. During this session, physiological data was collected. Several weeks later, physiological data was also gathered during a test. The Empatica E4 wearable sensor device was used throughout these sessions to continuously capture physiological signals.
Data format	Raw Preprocessed
Description of data collection	The procedure for collecting physiological data took place at intervals of one to two weeks between the start date of the midterm and the end date of the spring semester exams. The application period for the survey was scheduled to commence at least one week before the exam date. Considering that the minimum age for college enrollment is 17, the minimum age requirement for participants was established accordingly. The data was collected from university students in Istanbul, Turkiye, who were enrolled in various departments. Under the supervision of an expert psychologist in the field, 15-minute sessions were conducted to create a stressful environment. During these sessions, participants were intentionally exposed to stress, and their physiological signals were recorded using the Empatica E4 wristband, a wearable sensor. Participants were also instructed to report instances of tension or distress during the sessions. Emotional states were determined and labeled based on audio recordings captured during the sessions.
Data source location	Institution: Istanbul Kultur University, Department of Computer Engineering • City/Town/Region: İstanbul, Bakırköy • Country: Turkiye • Latitude and longitude (and GPS coordinates, if possible) for collected samples/data: 41° 5′ 7.3284′' N 29° 2′ 22.3836′' E
Data accessibility	Repository name: Mendeley Data Data identification number: 10.17632/z9y2fn38fp.1 Direct URL to data: https://data.mendeley.com/datasets/z9y2fn38fp/1

## Value of the Data

• To assess stress responses through physiological signals, data was extracted from the Empatica e4 wristband, a wearable sensor. Additionally, a survey was conducted using validated stress scales and questionnaires to gather general profile information.
• The dataset consists of survey responses from 48 individuals and physiological signals from 20 individuals in therapy and 8 individuals during exams.
• The dataset holds significant value for categorizing acute stress levels and analyzing physiological signals collected during exams or therapy sessions.
• The dataset can be utilized for various purposes, such as analyzing survey data and signals, training and testing models, validating findings, and refining the classification of stress levels based on physiological signals.

## 1 Objective

Stress is a common occurrence in people's daily lives, and based on scientific medical evidence, it is apparent that stress has both physiological and psychological effects on individuals [1]. It has repercussions on many facets of our lives, including educational [2,3] and occupational success [4]. Therefore, studying how stress affects performance is a vital area of study. The goal of this project is to advance this field by creating a dataset that can be used to examine the impact of stress on performance, including data from questionnaires and physiological measures. The objective of this research is to determine if there is a correlation between stress, as measured by the TOAD's stress scale [5], and other physiological signs. The study's secondary objective is to make available labeled signal datasets for use in analysis, classification, and prediction in related future studies. Overall, this study aims to contribute to our understanding of how stress affects performance and pave the way for other studies in this area.

## 2 Data Description

The proposed data repository contains 4 different file types:

• The dataset contains the “surveydataset.csv” file containing the survey answers of 48 participants. The data description for the survey dataset is shown in Table 1. The first ten features represent the participant's personal information. The next 34 questions are TOAD's stress scale.

**Table 1:** Survey dataset description.

Data	Data Type	Data Description
id	Integer	Participant id
age	Integer	Participant's age
gender	String	Participant's gender
gpa	String	Participant's GPA
grade	String	Indicates which grade the participant is in
anxiety history	String	Whether the participant has a history of anxiety or panic attacks
department	String	Participant's department
scholarship		Participant's scholarship rate.
graduate education plan	Boolean	Whether the participant has a master's plan
smoking habit	Boolean	Does the participant smoke?
regular medication	String	Does the participant use medication? If yes, what?
Q1-Q34	Integer	TOAD's Stress Scale (34 Questions)
score	Integer	Total point of Stress Scale
stress level	String	Class label according to points

In the survey dataset, 17.24% of male participants were observed to have high test anxiety while 30% of female participants appear to have high test anxiety. The Table 2 shows the distribution of male and female participants according to low, medium and high test anxiety.

• There are 28 different folders in the “physiological signals” folder. Each folder represents one participant. The folder name starting with “E” refers to the data collected at the time of the exam, and the remaining 20 folders starting with “T” indicate the data collected during the therapy sessions with the psychologist. These folders are created automatically in the Empatica e4 application. Each folder contains seven csv files, including “ACC, BVP, EDA, HR, IBI, tags, TEMP,” and one informational text file. The description of each signal is given in Table 3.

**Table 3:** Description of signals.

Data	Data Description
Blood Volume Pressure (BVP)	Participant's blood volume pressure
Electrodermal Activity (EDA)	Participant's electrodermal activity EDA expressed as microSiemens (μS)
Body Temperature (TEMP)	Participant's body temperature Body temperature expresses as Celcius (°C)
Accelerometer (ACC)	Participant's 3-axis accelerometer value (Continuous Gravitational Force (g))
Heart Rate (HR)	Participant's average heart rate in spans of 10 seconds.
Inter-beat interval (IBI)	The time interval between individual beats of the participant's heart.
• As seen in Fig. 1, the “downsample.csv” file contains the time, BVP, EDA, HR and ACC values of 103812 data. There are 55569 normal, 46646 stressed and 1597 upset labeled signals. Total of three labels were formed; 0 as normal, 1 as stressed, and 2 as upset.

**Fig. 1:**
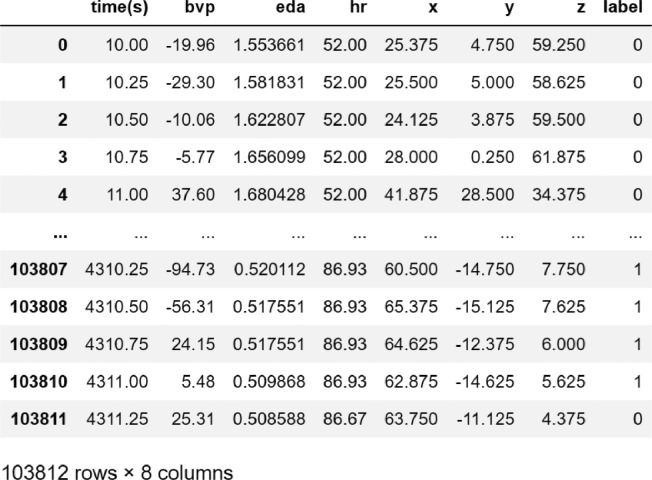
Samples of down-sampled dataset.
• The “upsample.csv” file contains a total of 1620978 labeled signals, of which 879742 are normal, 713295 are stressed and 27941 are upset. This file involves time, BVP, EDA, HR and 3-axis accelerometer signal data and labels with 3 classes (normal, stressed and upset).

**Table 2:** The distribution of male and female participants according to anxiety level.

Score	Definition	Study Subjects		
		Female	Male	Total
34-78	Low	1(5\%)	2(6.9\%)	3(6.12\%)
79-125	Normal	13(65\%)	21(75\%)	34(70.83\%)
126-170	High	6(30\%)	5(17.24\%)	11(22.45\%)
	Total	20	28	48

## 3 Experimental Design, Materials and Methods

### 3.1 Study Procedure

**Fig. 2:**
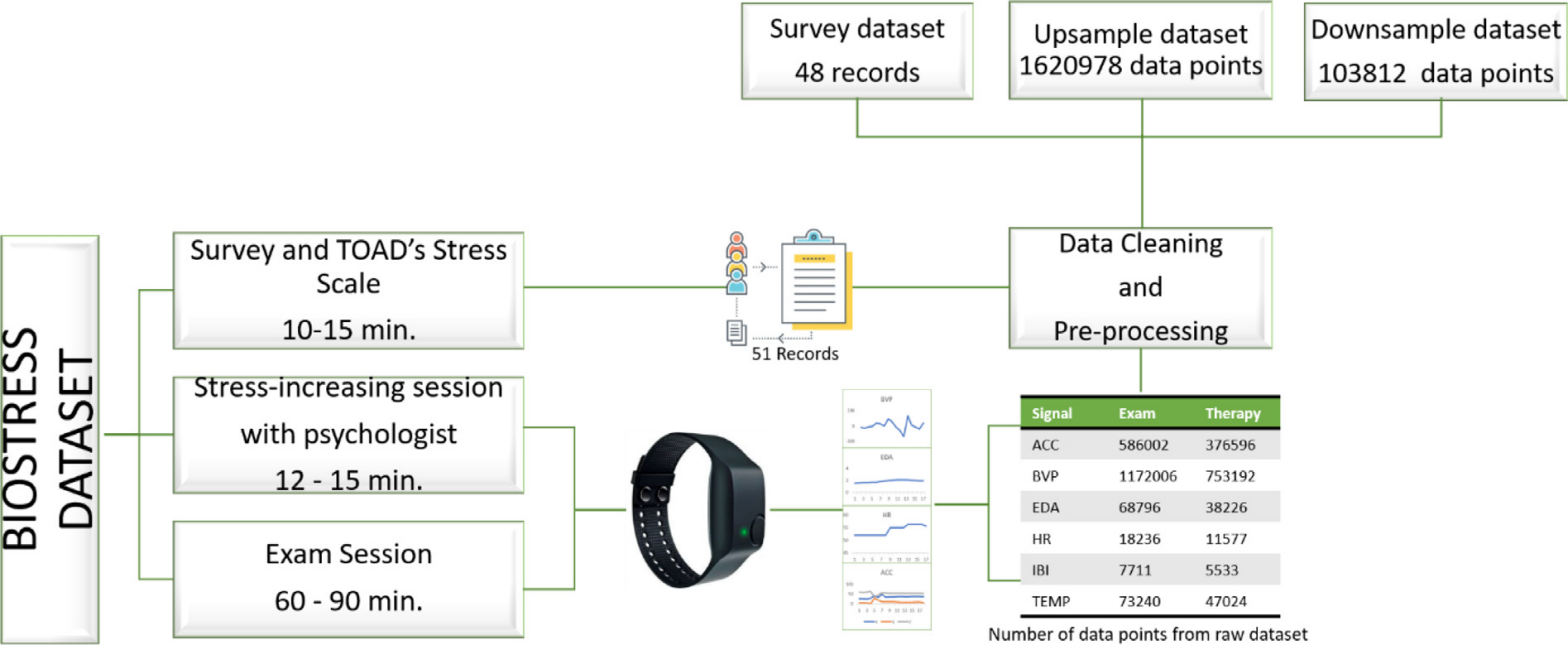
Procedures for acquiring and processing data.

The research was conducted with approval from the ethical committee of Istanbul Kultur University (decision dated 23 May 2022, number 2022.107). As can be seen in Fig. 2 collecting the data for BIOSTRESS involves three distinct steps. First, the participants were carefully selected by asking basic demographic questions like their age, level of anxiety, GPA, etc. The TOAD stress test followed, which took about 10 minutes. The participants were then exposed to a 15-minute exam stress-increasing session led by an expert psychologist in the field. The participants' biosignal data was gathered at this point. Some participants' signals were also gathered during the real exam, which took place a few weeks later than stress-increasing session. It takes about 60 minutes per participant, excluding exam time, to collect the data.

### 3.2 Study Population

**Fig. 3:**
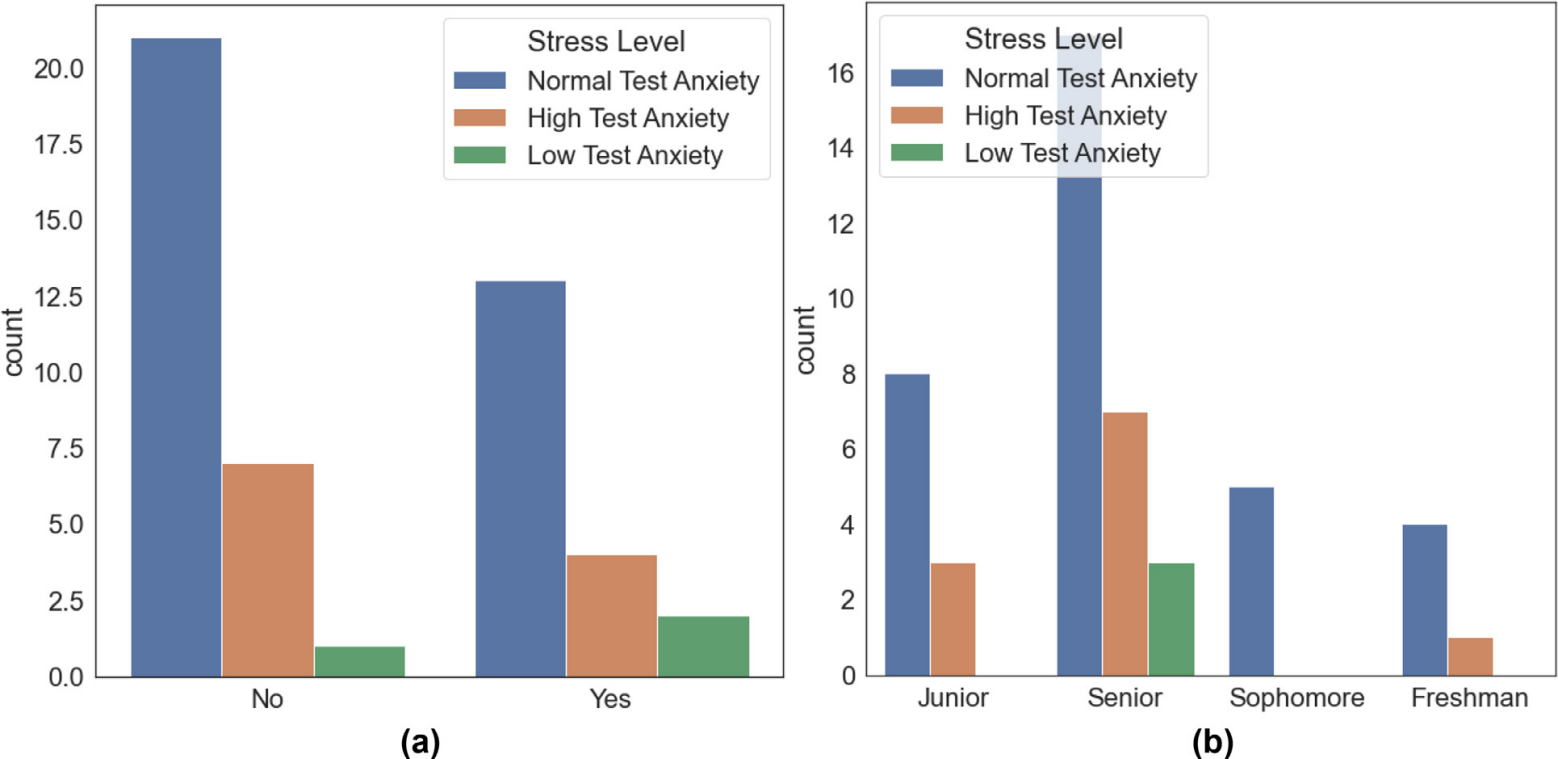
By stress level: a) Smoking habit b) Educational status.

In total, 51 people were asked to fill out a questionnaire that measured demographics and stress levels before meeting with a psychologist to collect physiological data. Twenty individuals committed to taking part in the sessions while a psychologist was present. All 20 participants were college students between the ages of 20 and 24, 7 of whom were female and 13 of whom were male. 88% of these people had never experienced anxiety before and 40% of the participants have a smoking habit. The distribution of the participants' educational status and smoking habits according to their stress levels is shown in Fig. 3. Participants are 27 seniors, 11 juniors, 5 sophomores, and 5 freshmen, and 28 of these students (58.33%) have a GPA between 2.00-2.99. 6 of the participants have a GPA above 3.

The department information of the participants is given in Table 4. Others includes child development, law, tourism management, business administration, biomedical engineering, and public finance departments. 60.42% of students have a plan to further their education by doing a master's degree.

**Table 4:** Departmental distribution of participants.

Department	Number of Participant
Computer Engineering	32
Accounting and Taxation	6
Architecture	2
Electrical and Electronics Engineering*	2
Others	6

⁎ An electrical and electronics engineering student is also a double major student with the computer engineering department.

### 3.3 The biosignal data

Continuous physiological data, including blood volume pressure (BVP), accelerometers (ACC), body temperature, and electrodermal activities (EDA), were collected using the Empatica e4 wristband. Non-adjustable parameters consist of sampling rates of 64 Hz for BVP, 4 Hz for EDA, 32 Hz for ACC, and 4 Hz for temperature signals. EDA and BVP signals are also considered to be associated with stress-related physiological signals [6], [7], [8], [9].

Additionally, the audio was recorded during the sessions. Simultaneously, the participants were instructed to indicate when they felt upset or anxious/stressed. Following that, physiological data were obtained during the exam. Exam duration may vary depending on the nature of the exam and the student's presence in the exam room. Nonetheless, the data collection session lasted at least 60 minutes. Based on these recordings, emotional states were determined and labeled.

### 3.4 The questionnaire data

In this research, the TOAD's Stress Scale has been used due to the fact that it handles the social, environmental, and academic aspects of exam anxiety. This questionnaire was created by adapting 50 anxiety statements developed in Baltas's study [10] to 34 questions. Reliability tests were conducted with 206 undergraduate students and the questionnaire was published. Originally in the dataset, there were 54 object features, an integer feature, and a float feature; 34 were TOAD's Stress Scale questions, 13 were personal questions, and the rest were confirmation questions. A total of 51 students completed the survey; three of the responses were eliminated for being incomplete or irrelevant. There were 28 males (58.33%) and 20 females (41.66%) aged 19 to 26 years, with a mean age of 22.31. In the form that we asked participants to fill out, there were two parts. The first part contains personal questions to get age, gender, GPA, grade of the student, anxiety history, department, scholarship, academic plan, smoking, and medication data, and the second part consists of TOAD's Stress Scale questions [Appendix A1].

### 3.5 Data Pre-processing

After obtaining the data, it was examined and preprocessing steps were implemented based on signal labeling and frequency. During the process of data cleansing, it was determined that three participants either did not complete the questionnaire or provided irrelevant responses. Consequently, these responses were omitted from the dataset. According to the TOAD Stress Scale, participants with a score between 34 and 78 are deemed to have “low test anxiety.” Those with scores between 79 and 125 are deemed to have “normal test anxiety,” while those with scores between 126 and 170 have “high test anxiety.” A “StressLevel” label class was created to record the determined stress levels of participants.

The first and last 10 seconds of the data received during the session are meeting-session outcome components, so the activities were omitted from the dataset. Due to the variable frequency of the data, resampling based on the nearest value was also performed. Throughout this procedure, downsampling and upsampling were applied to all data collected from each individual participant. Downsampling reduces the frequency of the data to 4 Hz, which is the frequency of the EDA. The data frequency is increased by upsampling to 64 Hz, which is the BVP frequency. There are 103812 float data samples in the downsampled dataset, while there are 1620978 float data samples in the upsampled dataset.

**Fig. 4:**
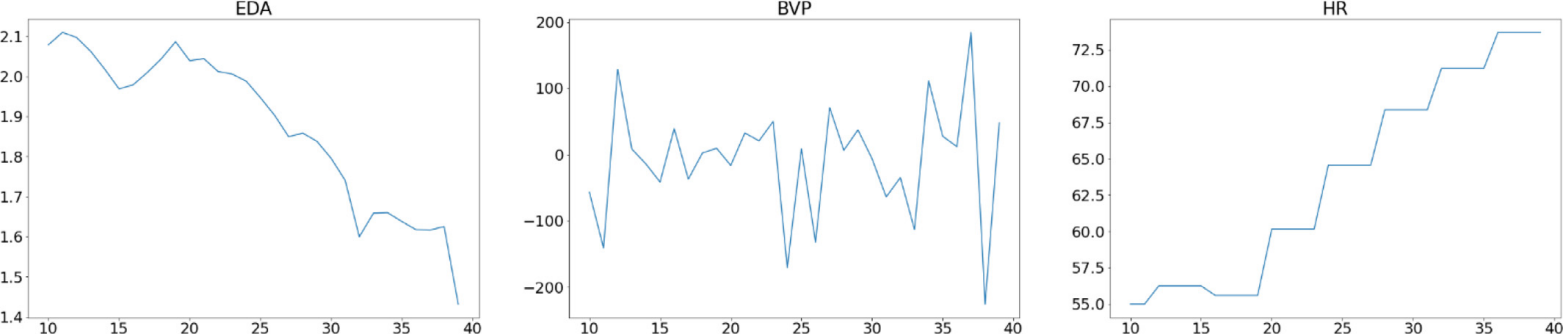
Downsampled data sample of thirty seconds.

**Fig. 5:**
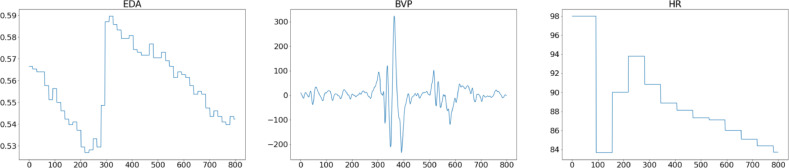
Upsampled data sample of thirty seconds.

The beginning and finishing positions of the labeling were selected using the Label Studio [11] interface. Tags were constructed using audio recordings of participants during the psychological session, and tagging was done using 30-second windows of time-serial data in an e4 Connect interface. The first 30 seconds of sampled data for downsampled and upsampled signals are depicted in Figs. 4 and 5, respectively. In both downsampled and upsampled datasets, 0 represents normal, 1 stressed, and 2 upset labels. Comparing these figures reveals that the peak intensity as a function of time decreases due to the fact that the data received at 64 Hz for the BVP signal is reduced to 4 Hz in the downsampled dataset.

**Fig. 6:**
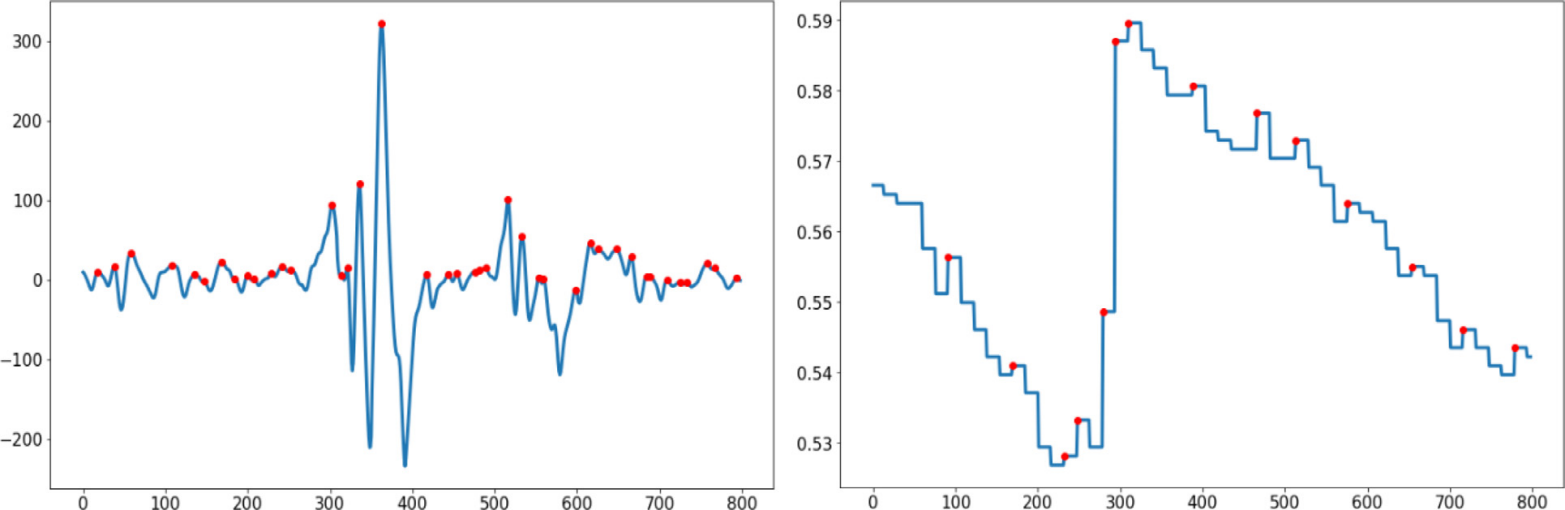
30-seconds Upsampled Data Sample from the BVP&EDA signals.

**Fig. 7:**
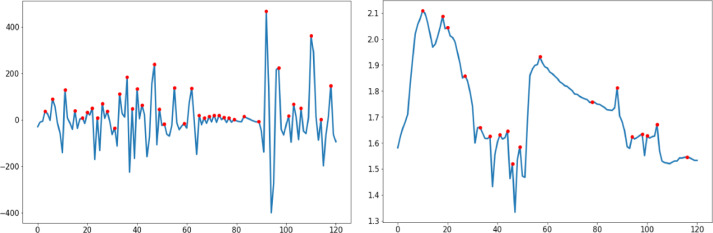
30-seconds Downsampled Data Sample from the BVP&EDA signals.

The peaks are depicted in Figs. 6 and 7 by the red dots, whereas the original plot is shown by the blue lines. In addition to the peak points that have been recognized, the gathered data and anomalous graph alterations can be independently assessed for the labels of normal, stressed and upset. When compared to the intervals that came before and after the indices 300 to 400, both BVP and EDA display an abnormal variance. As a direct consequence of this, signals can be connected to one another.

## Ethics Statements

Within the scope of the study, data were collected with the permission of the ethics committee of Istanbul Kultur University, with the decision dated 23.05.2022 and numbered 2022.107.

## CRediT authorship contribution statement

**Çağla Çöpürkaya:** Data curation, Visualization, Investigation. **Elif Meriç:** Data curation, Visualization, Investigation. **Elif Berra Erik:** Data curation, Visualization, Investigation. **Büşra Kocaçınar:** Data curation, Visualization, Investigation, Writing – original draft, Writing – review & editing. **Fatma Patlar Akbulut:** Supervision, Conceptualization, Methodology, Investigation, Writing – review & editing. **Cagatay Catal:** Investigation, Writing – review & editing.

## Declaration of Competing Interest

The authors declare that they have no known competing financial interests or personal relationships that could have appeared to influence the work reported in this paper.

## Data Availability

BIOSTRESS (Original data) (Mendeley Data).

## Appendix Supplementary materials

Image, application 1
