# Supplementary material for: Investigating the effects of stress on achievement: BIOSTRESS dataset
Source: Data Brief. 2023 Jun 8;49:109297. doi: 10.1016/j.dib.2023.109297 (PMC10279542; doi:10.1016/j.dib.2023.109297)
Supplement: Supplementary file 1 [file mmc1.docx]

Appendix A1: **Exam/Test Anxiety Scale**

| Please choose the most appropriate response. | Never | Rarely | Sometimes | Often | Always |
| --- | --- | --- | --- | --- | --- |
| - I wish there was a method to successfully complete the course without taking the exam. | 1 | 2 | 3 | 4 | 5 |
| - The people who care about me (family, friends) count on me to do well academically. | 1 | 2 | 3 | 4 | 5 |
| - Whenever I have tests or exams, my mind tends to wander and I become mentally distracted. | 1 | 2 | 3 | 4 | 5 |
| - Exams need not necessarily be solemn, stressful, and nerve-wracking occasions. | 1 | 2 | 3 | 4 | 5 |
| - Before or after significant exams, I have no appetite. | 1 | 2 | 3 | 4 | 5 |
| - If there were no exams, I would have learned the material more effectively. | 1 | 2 | 3 | 4 | 5 |
| - My anxiety about performing well on exams negatively affects my study practices and performance. | 1 | 2 | 3 | 4 | 5 |
| - When I have a significant exam approaching, I am unable to sleep well. | 1 | 2 | 3 | 4 | 5 |
| - I worry about other people's opinions of me when I get a bad grade. | 1 | 2 | 3 | 4 | 5 |
| - If I failed the exams, my competence would be questioned by those around me. | 1 | 2 | 3 | 4 | 5 |
| - I never relax before the exams. | 1 | 2 | 3 | 4 | 5 |
| - Before critical exams, my brain often goes blank. | 1 | 2 | 3 | 4 | 5 |
| - Before an exam, I am always restless, tense, and anxious. | 1 | 2 | 3 | 4 | 5 |
| - We shouldn't base our future goals on how well we perform on exams. | 1 | 2 | 3 | 4 | 5 |
| - Exams do not reveal a person's true level of knowledge. | 1 | 2 | 3 | 4 | 5 |
| - When I get a poor grade, I never share it with anyone. | 1 | 2 | 3 | 4 | 5 |
| - When preparing for major exams, I have a tendency to think negatively and be anxious that I will perform poorly. | 1 | 2 | 3 | 4 | 5 |
| - Before the exam results are revealed, I feel anxious and uneasy. | 1 | 2 | 3 | 4 | 5 |
| - I prefer not to be required to take exams when applying for a job. | 1 | 2 | 3 | 4 | 5 |
| - Exam failure makes me doubt my intelligence. | 1 | 2 | 3 | 4 | 5 |
| - My exam anxiety prevents me from being fully prepared for the exam, thereby increasing my tension. | 1 | 2 | 3 | 4 | 5 |
| - I recognize that I shake my leg and tap my fingers on the bench during the exam. | 1 | 2 | 3 | 4 | 5 |
| - After the exams, I usually think I could have done better. | 1 | 2 | 3 | 4 | 5 |
| - I underperform in exams because my emotions distract me. | 1 | 2 | 3 | 4 | 5 |
| - My self-confidence suffers whenever I fail. | 1 | 2 | 3 | 4 | 5 |
| - Certain muscles in my body constrict during exams. | 1 | 2 | 3 | 4 | 5 |
| - Before exams, neither can I completely rely on me nor can I relax. | 1 | 2 | 3 | 4 | 5 |
| - If I failed the exam, I would lose the favor of my friends. | 1 | 2 | 3 | 4 | 5 |
| - Not knowing if I am adequately prepared for an exam is one of my main issues. | 1 | 2 | 3 | 4 | 5 |
| - When taking extremely crucial exams, I experience physical panic. | 1 | 2 | 3 | 4 | 5 |
| - Teachers/professors should consider students' excitement into account when evaluating an exam. | 1 | 2 | 3 | 4 | 5 |
| - I'd like to know my friends' grades before revealing mine. | 1 | 2 | 3 | 4 | 5 |
| - It concerns me that some of my acquaintances will make fun of me if I receive a poor grade. | 1 | 2 | 3 | 4 | 5 |
| - During exams, I often become overly excited and forget what I actually know. | 1 | 2 | 3 | 4 | 5 |
